# Recent Studies of Membranes for Liquids Separation and Water Treatment

**DOI:** 10.3390/membranes13090779

**Published:** 2023-09-04

**Authors:** Mohammadamin Ezazi, M. M. Quazi, Hossein Taheri

**Affiliations:** 1 Department of Mechanical Engineering, Georgia Southern University, Statesboro, GA 30460, USA; 2 Faculty of Mechanical and Automotive Engineering Technology, Universiti Malaysia Pahang, Pekan 26600, Pahang, Malaysia; moinuddin@ump.edu.my; 3 Department of Manufacturing Engineering, Georgia Southern University, Statesboro, GA 30460, USA; htaheri@georgiasouthern.edu

Rapid urbanization and industrialization in the past decades have resulted in vast amounts of wastewater containing pollutants such as inorganic chemicals, pathogens, pharmaceuticals, plant nutrients, petrochemical products, and microplastics [1,2,3]. The extensive discharge of wastewater into the environment without proper treatment poses unprecedented threats to humans, marine life, and other organisms [4,5]. Remediating and separating these large wastewater bodies requires methods that enable fast operation and high separation efficiency with a relatively low energy consumption. Membrane-based liquid separation and water treatment technologies can meet these requirements as they rely on relatively low thermal inputs and additives while providing promising permeation flux and separation efficiency [6,7,8,9,10,11,12,13].

This Special Issue titled “Recent Studies of Membranes for Liquids Separation and Water Treatment” emphasizes different aspects of the membranes employed in separating liquid mixtures and wastewater remediation. These aspects include materials, synthesis and fabrication, separation mechanisms, performance evaluation, modeling and simulations, and applications.

Desalinating saline water has proven to be an effective method to meet the escalating demand for fresh drinking water. Among various desalination technologies, vacuum membrane distillation is a relatively cost-effective method that can enable a high water vapor flux under sufficiently high vacuum levels [14,15]. In a study by Idrees et al., techno-economic analyses were conducted on vacuum membrane distillation for removing NaCl and KCl salts from seawater [16]. The lab-scale experiments were conducted utilizing a polytetrafluoroethylene (PTFE) membrane under a variation of different parameters, including temperature, concentration, pressure, and velocity [16]. Subsequently, economic investigations were performed based on the experimental results in combination with a market survey to estimate the costs of an up-scale vacuum membrane distillation plant. The results indicated that the costs associated with obtaining clean water using lab-scale vacuum membrane distillation can be reduced by utilizing a large-scale setup [16].

Forward osmosis is another prominent water treatment process that relies on the difference in osmotic pressure induced by different concentrations of the feed and draw solutions [17]. In a study by Mendoza et al., the forward osmosis technique was implemented using magnesium phosphate salts recovered from wastewater as the draw solution [18]. Nitric acid and citric acid were utilized to facilitate the dissolution of salts, including cattiite, struvite, and hazenite, as they show limited solubility in water. This setup enabled water extraction to produce nutrient solutions [18]. Subsequently, the nutrient solutions were supplied as fertilizers to plants in a hydroponic system. The results of the study showed the feasibility of functional growth of lettuce by utilizing recovered water as a source of nutrition [18].

The permeation flux in the forward osmosis process is relatively lower compared to that in the membrane-based separation processes that operate under pressure [19,20]. To achieve an acceptable permeation flux in forward osmosis setups, a better understanding of the parameters affecting the flux is necessary, such as the interactions between the solvent and solute. Higuchi et al. utilized the non-equilibrium molecular dynamics (NEMD) method to quantitatively investigate the permeation flux through a semi-permeable forward osmosis membrane under different solvent–solute interatomic interactions [20]. The calculated permeation flux results were consistent with the theoretical curves obtained from the combination of the permeation flux and Van’t Hoff equations [20]. The results also validated the NEMD method for evaluating permeation flux in a forward osmosis setup [20].

The release of pharmaceutical and personal care products (PPCPs) such as carbamazepine (CBZ) from conventional wastewater treatment facilities to the environment is another challenge that requires immediate attention, as these products can have detrimental effects on health even at low concentrations [21,22]. In a study by Dao et al., a moving-bed membrane bioreactor system combined with an electrochemical process was implemented to remove persistent CBZ and phosphate pollutants from synthetic hospital wastewater [22]. The results implied that using a moving-bed membrane bioreactor could effectively remove components such as ammonia, whereas it could not effectively remove CBZ and phosphate. A factorial design was subsequently applied to optimize the process and determine the optimum operating conditions for the electrochemical process to improve the removal of CBZ and phosphate [22]. The results indicated that the electrochemical method as an oxidation process can be effectively combined with a moving-bed membrane bioreactor to remediate wastewater containing CBZ and phosphate [22].

The contamination of water bodies by oil and other organic contaminants represents another environmental hazard with potential impacts not only on humans but also on marine and terrestrial ecosystems [23,24,25]. A review by Ezazi et al. summarized the recent developments in oil–water separation membranes modified with two-dimensional (2D) materials [26]. In particular, the review focused on the membranes modified with synthetic 2D materials, including graphene family materials, MXenes, metal–organic frameworks (MOF), and covalent organic frameworks (COF). The synthesis and fabrication methods, wettability, permeation flux, separation efficiency, and the type of oils separated by these membranes were summarized [26]. The 2D materials with uniform pore size, high surface area, tunable wettability, and relatively high thermal and chemical stability were found to be effective alternatives to the current materials for developing membranes with promising separation efficiency and flux. Further, these unique properties of the membranes modified with 2D materials make them highly applicable for the sustainable treatment of complex industrial wastewater [26]. Despite these advantages, this review encourages further studies on different aspects of 2D materials-based oil–water separation membranes, including thickness and durability, cytotoxicity, long-term stability, incorporating additional functionalities, and large-scale fabrication [26].

Structural integrity is a crucial attribute of membranes that can affect separation performance. Manufacturing imperfections, membrane aging and degradation, and membrane fouling can deteriorate separation performance, resulting in improper liquid separation or water treatment [27,28]. To ensure high-efficiency operation, the membranes should be continuously monitored and inspected to detect potential performance and physical degradation. However, performance evaluation may require disassembling membrane units [29], which can impose process delays and additional maintenance costs. Real-time monitoring can address this challenge by offering a continuous evaluation of membrane performance. Various methods have been proposed to monitor the integrity of membranes, such as acoustic sensor analysis, particle counting, and surrogate challenge tests [30]. With the increasing demand for membranes for various applications, developing new real-time monitoring and detection techniques is crucial. In particular, the response time, simplicity, accuracy, continuity, and cost-effectiveness are among the factors that should be considered when developing new membrane monitoring systems [30].

In summary, the contributions featured here cover a wide range of attributes and analysis methods for liquid separation and water treatment membranes, including membrane configurations, membrane materials, fabrication methods, type of contaminants removed, recovery potentials, optimizations to determine optimum operating conditions, and techno-economic analyses. These findings highlight the importance of fundamental studies and continuous research on membranes to develop new sustainable systems with higher efficiency and lower energy requirements that can be applied to treat a wide range of liquid mixtures and wastewater bodies.
